# Calcium influx, oxidative stress, and apoptosis induced by TRPV1 in chronic myeloid leukemia cells: Synergistic effects with imatinib

**DOI:** 10.3389/fmolb.2023.1129202

**Published:** 2023-02-15

**Authors:** Federica Maggi, Maria Beatrice Morelli, Cristina Aguzzi, Laura Zeppa, Massimo Nabissi, Carlo Polidori, Giorgio Santoni, Consuelo Amantini

**Affiliations:** ^1^ School of Biosciences and Veterinary Medicine, University of Camerino, Camerino, Italy; ^2^ School of Pharmacy, University of Camerino, Camerino, Italy

**Keywords:** TRPV1, N-oleoyl-dopamine, calcium flux, chronic myeloid leukemia, oxidative stress, imatinib

## Abstract

**Introduction:** Calcium flux is the master second messenger that influences the proliferation–apoptosis balance. The ability of calcium flux alterations to reduce cell growth makes ion channels interesting targets for therapy. Among all, we focused on transient receptor potential vanilloid 1, a ligand-gated cation channel with selectivity for calcium. Its involvement in hematological malignancies is poorly investigated, especially in the field of chronic myeloid leukemia, a malignancy characterized by the accumulation of immature cells.

**Methods:** FACS analysis, Western blot analysis, gene silencing, and cell viability assay were performed to investigate the activation of transient receptor potential vanilloid 1, by N-oleoyl-dopamine, in chronic myeloid leukemia cell lines.

**Results:** We demonstrated that the triggering of transient receptor potential vanilloid 1 inhibits cell growth and promotes apoptosis of chronic myeloid leukemia cells. Its activation induced calcium influx, oxidative stress, ER stress, mitochondria dysfunction, and caspase activation. Interestingly, a synergistic effect exerted by N-oleoyl-dopamine and the standard drug imatinib was found.

**Conclusion:** Overall, our results support that transient receptor potential vanilloid 1 activation could be a promising strategy to enhance conventional therapy and improve the management of chronic myeloid leukemia.

## 1 Introduction

Intracellular calcium (Ca^2+^), one of the most important second messengers, is subjected to continuous fluctuations, essential in the control of cell proliferation, differentiation, migration, and death (Cui et al., 2017; Zhang et al., 2017; Bootman et al., 2018; Giorgi et al., 2018). Interestingly, it is well recognized that the irregularity in Ca^2+^ channel expression and functions and the consequent associated alterations of the Ca^2+^ flux contribute significantly to the development of cancer. It is therefore not surprising that in recent years, cancer has also been classified as a “channelopathy” (Litan and Langhans, 2015). In this scenario, several studies described the essential role played by transient receptor potential (TRP) channels, the non-selective cation-permeable receptors localized in plasma membrane, endoplasmic reticulum, mitochondria, and lysosomes, in regulating cation concentrations and membrane voltage (Gees et al., 2010). Furthermore, many scientific contributions described the involvement of TRP dysregulations in cancer growth, metastasis, and chemoresistance (Shapovalov et al., 2016). Among TRP channels, the opening of TRP vanilloid 1 (TRPV1), a ligand-gated cation channel activated by capsaicin (CPS), resiniferatoxin, temperatures over 43°C, acidic conditions (pH < 6), and endogenous cannabinoids, impacts the fine regulation of the proliferative signaling/cell death pathways by mainly modifying the Ca^2+^ influx (Zhai et al., 2020). TRPV1 activation was found to be involved in the induction of cell death in different cancer models including bladder cancer (Amantini et al., 2009), glioma (Amantini et al., 2007), human gastric cancer, and breast cancer (Pecze et al., 2016; Liu et al., 2022). Moreover, the triggering of TRPV1 facilitates the stimulation of pro-apoptotic and anti-proliferative effects in primary lymphoblasts obtained from both patients with T-acute lymphoblastic leukemia and the Jurkat cell line (Punzo et al., 2018). In this regard, the involvement of TRP channels in hematological malignancies is poorly investigated, especially in the field of myeloid leukemia. In this tumor type, it has only been demonstrated that the activation of TRPV5 and TRPV6 promotes a Ca^2+^-dependent cell cycle arrest (Heise et al., 2010), and the inhibition of TRPM7 impairs cell proliferation and stimulates erythroid differentiation in K562 cells (Takahashi et al., 2018). In addition, we recently showed that TRPV2 activation induced mitophagy associated with cell growth inhibition and stemness reduction in chronic myeloid leukemia (CML) cells (Maggi et al., 2022).

CML is a hematological malignancy that is characterized by the accumulation of immature cells as the consequence of the inadequate differentiation pathway of the hematopoietic progenitors. The evolution of this disease comprises three stages: chronic phase, accelerated phase, and blast crisis (Osman and Deininger, 2021). The present-day approved therapy is the administration of tyrosine kinase inhibitors (TKIs) in addition to allogeneic stem cell transplantation or interferon administration. However, the prolonged treatment with TKIs leads to the development of resistance and, for this reason, therapy interruption is required which affects the survival of patients (Apperley, 2015). Therefore, considering the implications of using TRPV1 modulators as potential therapeutic approaches represents a fascinating challenge and a new resource in the pharmacological field. To date, two dozen TRPV1 modulators are already used in clinical studies to evaluate their effects in inflammation and pain (Li et al., 2021). The historical agonists of the TRPV1 channels are CPS and resiniferatoxin, but in the last decades a new, more selective, and potent agonist, N-oleoyl-dopamine (OLDA), has attracted interest (Chu et al., 2003).

Thus, the aim of this study is to investigate the activation of TRPV1 by OLDA in CML cells, focusing the attention on signaling pathways such as autophagy, endoplasmic reticulum (ER) stress, and apoptotic cell death. Furthermore, the co-administration of OLDA with the conventional drug imatinib was also investigated to elucidate the potential role of the combination of these two drugs in managing CML.

## 2 Materials and methods

### 2.1 Cell lines

Human chronic myeloid leukemia K562, KU812, and MOLM-6 cell lines were obtained from DSMZ-German Collection of Microorganisms and Cell Cultures GmbH (DSMZ, Braunschweig, Germany) and were maintained in RPMI-1640 medium (Euroclone Ltd., Devon, United Kingdom) supplemented with 10% heat-inactivated fetal calf serum (Euroclone), 2 mM L-glutamine, 100 IU/ml of penicillin, and 100 μg/ml of streptomycin.

The buffy coat, no longer used for transfusion, kindly provided by the Transfusion Center of Macerata Hospital after authorization of the hospital management (Direzione Medica Presidio Ospedaliero Unico AV3, Medical Director Dr. Carlo Di Falco), was only used *in vitro* to obtain an enrichment of normal myeloid cells using a RosetteSep^TM^ HLA Myeloid Cell Enrichment Kit (STEMCELL Technologies, Cambridge, United Kingdom).

### 2.2 Chemicals and reagents

Capsazepine (CPZ), capsaicin (CPS), imatinib mesylate, ionomycin, propidium iodide (PI), and 2′,7′-dichlorofluorescin diacetate (DCFDA) were purchased from Sigma-Aldrich (Milan, Italy). The Fluo-3 AM calcium indicator and JC-1 were purchased from Thermo Fisher Scientific (Waltham, MA, United States). A784168 was purchased from Bio-Techne S.R.L (Milan, Italy). N-oleoyl-dopamine (OLDA) was purchased from Tocris (Bristol, United Kingdom). CPZ, CPS, and OLDA were dissolved in DMSO and were used as vehicle (maximum percentage 0.05, considered non-toxic) (de Abreu Costa et al., 2017).

Antibodies (Abs) were used according to manufacturer’s instructions: anti-phospho-histone γH2AX (Ser139) (γH2AX), anti-caspase 3, anti-binding immunoglobulin protein (BiP), anti-phospho-ubiquitin (pSer65), anti-microtubule-associated protein-1 light chain 3 (LC3), anti-activating transcription factor 4 (ATF4), anti-autophagy protein 12 (ATG5–ATG12), and anti-GAPDH were purchased from Cell Signaling Technology (1:1000, Danvers, MA, United States). Anti-transient receptor potential channel vanilloid 1 (TRPV1) was purchased from Invitrogen (1:1000, Waltham, Massachusetts, United States). Secondary Abs used were: HRP-conjugated anti-rabbit IgG (1:5000, Jackson ImmunoResearch Europe Ltd., Ely, United Kingdom); HRP-conjugated anti-mouse IgG (1:2000, Cell Signaling Technology); and PE-conjugated goat anti-rabbit Ab (1:40, BD Biosciences, Milan, Italy). The OxyBlot Protein Oxidation Detection Kit was purchased from Merck Life Science (Milan, Italy).

### 2.3 Cell viability assay

A density of 2 × 10^5^ CML cells/mL was plated in 12-well plates. The next day, cells were exposed to different concentrations of OLDA (0.5–100 μM), CPS (10–300 μM), or the respective vehicle for 24 h. Then, cells were stained with trypan blue and counted using the TC20 automated cell counter according to the instrument instruction (Bio-Rad, Milan, Italy). This automated cell counter uses multifocal plane analysis to assess cell viability. Three replicates were carried out for each treatment. IC_50_ (half-maximal inhibitory concentration) values, shown as the mean ± standard deviation (SD), were calculated using GraphPad Prism^®^ 9.1 (GraphPad Software, San Diego, CA, United States). In some experiments, cell counting was performed on siTRPV1 or siGLO CML cells treated with OLDA (IC_50_) or vehicle. OLDA was used in combination with imatinib mesylate for 24 h. Synergistic activity of OLDA–imatinib combination was determined by isobologram analysis and combination index (CI) methods (CompuSyn Software, ComboSyn, Inc.). The CI was used to express synergism (CI < 1), additivity (CI = 1), or antagonism (CI > 1).

### 2.4 Gene silencing and quantitative real-time PCR (qRT-PCR)

TRPV1 (siTRPV1) and siGLO non-targeting siRNA (used as the control), FlexiTube siRNA, were purchased from Qiagen (Milan, Italy). The day before transfection, CML cells were diluted at the density of 6 × 10^5^ cells/mL in the culture medium. After 24 h, cells were collected, counted, and plated at the density of 4 × 10^5^ cells/mL, and siTRPV1 or siGLO (50 nM) was added according to the HiPerFect Transfection Reagent protocol (Qiagen). Cells were then harvested at 48 h post transfection. Silencing efficiency was evaluated by qRT-PCR and Western blotting. No differences in TRPV1 expression and cell viability were observed in siGLO-transfected cells compared with siGLO-untransfected cells.

Total RNA was extracted using the RNeasy Mini Kit (Qiagen), and cDNA was synthesized using the iScript Advanced cDNA Synthesis Kit (Bio-Rad) according to manufacturers’ protocol. qRT-PCR was performed using QuantiTect Primer Assays for human TRPV1 (QT00046109) and GAPDH (QT00079247), as a reference gene (Qiagen), using the iQ5 Multicolor Real-Time PCR Detection System (Bio-Rad). The PCR parameters were in accordance with the primer datasheet. All samples were assayed in triplicate. Gene expression analysis was performed using iQ5 software.

### 2.5 Intracellular calcium influx [Ca^2+^]_i_

Intracellular Ca^2+^ influx was measured using Fluo-3 AM and FACS analysis. Briefly, 1.5 × 10^6^ CML cells/mL were first washed in calcium- and magnesium-free PBS supplemented with 4.5 g/L of glucose and then incubated in calcium- and magnesium-free PBS/glucose medium supplemented with 7 μmol/L Fluo-3 AM for 30 min in the dark at 37°C and 5% CO_2_. After washing, cells were resuspended in calcium- and magnesium-free PBS/glucose medium containing 2 mmol/L Ca^2+^ and were stimulated with OLDA (IC_50_ dose) or with vehicle up to 3 min. In some experiments, CML cells, loaded as described previously, were treated with OLDA in combination with CPZ (10 µM) or A784168 (1 µM). Ionomycin (5 μg/ml) treatment was used as a positive control for measuring calcium influx. Fluo-3 AM fluorescence was measured using the BD Accuri C6 Plus flow cytometer and its software (Beckton Dickinson, San Jose, CA, United States).

### 2.6 Western blot analysis

Lysate from CML cells, treated or not treated with OLDA at the IC_50_ dose, was extracted using lysis buffer (10 mM Tris; 100 mM NaCl; 1 mM EDTA; 1 mM EGTA; 1 mM NaF; 20 mM Na_4_P_2_O_7_; 2 mM Na_3_VO_4_; 1% Triton X-100; 10% glycerol; 0.1% SDS; 0.5% deoxycholate; and 1 mM PMSF) containing protease-inhibitor cocktail (Euroclone). Proteins were separated on 8%–14% SDS-polyacrylamide gels and transferred using Bio-Rad systems. Non-specific binding sites were blocked with 5% low-fat dry milk or 5% BSA in PBS containing 0.1% Tween 20 for 1 h at room temperature. Membranes were incubated overnight at 4°C with anti-LC3, anti-ATG12, anti-ATF4, anti-BiP, anti-γH2AX, anti-caspase 3, anti-phospho-ubiquitin (pSer65), or anti-GAPDH Abs, followed by corresponding HRP-conjugated secondary Abs. In some experiments, Western blot analysis was performed on siTRPV1 and siGLO (control) CML cells treated with OLDA or vehicle to assess BiP expression levels. Moreover, lysates from CML cells were treated with OLDA at the IC_50_ dose and imatinib mesylate (0.5 µM), either alone or in combination, for 24 h were incubated with anti-γH2AX and anti-caspase 3.

In addition, the protein oxidation products were identified in CML cells, which were treated as described previously, by scanning carbonyl groups using the OxyBlot^TM^ Protein Oxidation Detection Kit according to the manufacturer’s instructions. In brief, dinitrophenylhydrazine was added to the crude total proteins (20 μg) to derive the carbonyl groups from the protein side chains. Carbonylated proteins were resolved by SDS-polyacrylamide gel electrophoresis, and Western blot analysis was performed using the provided anti-DNP antibody (1:150).

The detection was performed using LiteAblot PLUS kit, ChemiDoc, and Quantity One software (Bio-Rad). GAPDH was used as the loading control. SHARPMASS VI–VII (Euroclone) and SeeBlue Plus2 (Invitrogen) were used as pre-stained protein markers.

### 2.7 Cell death, reactive oxygen species (ROS) production, and mitochondrial transmembrane potential (∆Ψm) analysis

CML cells, treated with OLDA or vehicle at the IC_50_ dose for 24 h, were incubated with 2 μg/mL PI for 30 min at 37°C. After washing, the fluorescence intensity was analyzed using BD Accuri C6 Plus software. The fluorescent probe DCFDA was used to assess oxidative stress levels in siTRPV1 and siGLO CML cells after treatment with OLDA (IC_50_). Cells were incubated with 20 μM DCFDA for 20 min prior to the harvest time point. After washing, the fluorescence was assayed using the BD Accuri C6 Plus flow cytometer and its software. ∆Ψm was evaluated by JC-1 staining according to the manufacturer’s protocol in CML cells, treated with OLDA or vehicle at the IC_50_ dose for 24 h. Samples were then analyzed using the BD Accuri C6 Plus flow cytometer and its software.

### 2.8 Bioinformatics analysis

BloodSpot, Stemformatics, and GEO are open-access downloaded bio-database that provide visualization and are analyzing tools for large-scale genomics datasets. In particular, BloodSpot (https://www.bloodspot.eu) provides gene expression profiles of healthy and malignant hematopoiesis in humans or mice, encompassing a total of more than 5,000 samples analyzed using a oligonucleotide microarray chip and by RNA-seq assay (Bagger et al., 2016). Stemformatics (https://www.stemformatics.org/) is an established gene expression data portal containing over 420 public gene expression datasets derived from microarray, RNA sequencing, and single-cell profiling technologies. Its major focus is on pluripotency, tissue stem cells, and staged differentiation (Choi et al., 2019). The Gene Expression Omnibus database (http://www.ncbi.nlm.nih.gov/geo) is an open functional genomics database of a high-throughput resource (Barrett, 2004).

Analysis of data from database IDs 6326 and 6610 from Stemformatics (accessed on 4 November 2022) and GSE24759 and GSE13159 from GEO was performed *in silico* (accessed on 13 December 2022). The hierarchical tree was analyzed in the BloodSpot online database (accessed on 13 December 2022).

### 2.9 Statistical analysis

The statistical significance was determined by Student’s *t*-test and by ANOVA with Dunnett’s *post hoc* test. No statistically significant differences were found between siGLO-untransfected and -transfected CML cells, treated or not treated with a vehicle at different times (data not shown). Given the absence of differences, for simplicity, as the control, in time-course analysis, cells treated with vehicle for 24 h were shown.

## 3 Results

### 3.1 The activation of TRPV1 affects CML cell viability

We recently demonstrated the expression of TRPV1 on K562, KU812, and MOLM-6 CML cell lines, common myeloid progenitors, normal myeloid cell enrichment, and PBMCs, respectively (Maggi et al., 2022). To enhance our analysis of TRPV1 expression, we have also performed *in silico* analyses with datasets 6326/6610 from the Stemformatics database and GSE13159 from the GEO repository, demonstrating its expression in all types of leukemia cells (Supplementary Figure S1A). Interestingly, TRPV1 expression in common myeloid CML progenitors is modulated during the different phases of the disease with higher levels during the chronic phase (Supplementary Figure S1B).

**FIGURE 1:**
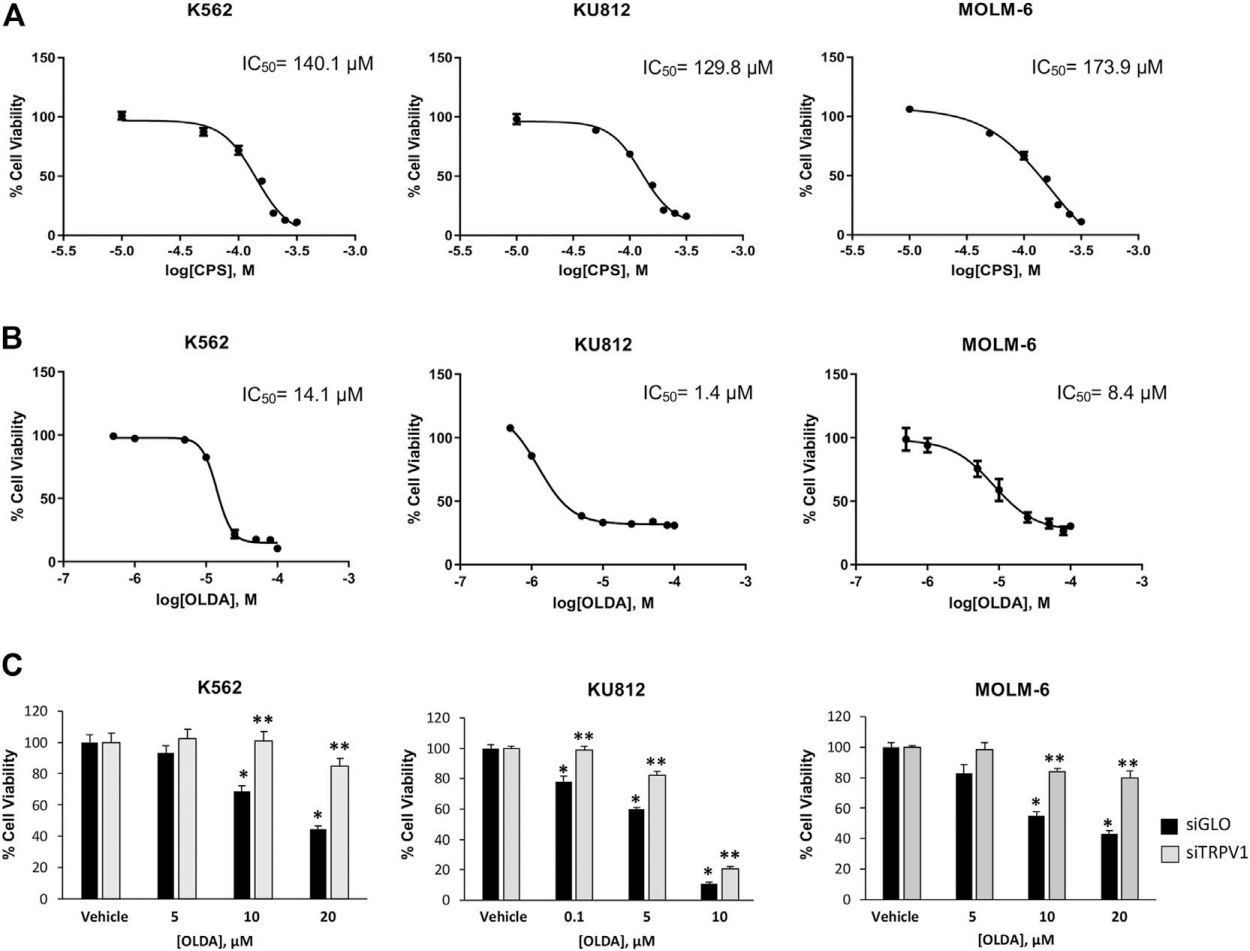
OLDA, the agonist of TRPV1, inhibits cell growth in CML cells. **(A)** Cell viability of CML cells treated for 24 h with different doses of CPS. Data are the mean ± SD for three experiments. **(B)** Cell viability of CML cells treated for 24 h with different doses of OLDA. Data are the mean ± SD of three experiments. **(C)** Cell viability was assessed in siTRPV1 and siGLO cells treated for 24 h with different doses of OLDA in the range of IC_50_ or the vehicle. Data are the mean ± SD of three experiments. *p < 0.05 vs the vehicle; **p < 0.01 vs OLDA-treated siGLO cells.

It is well known that TRPV1 is the receptor for CPS; however, several sources of evidence demonstrated that the endogenous compound OLDA is a more potent and selective TRPV1 agonist (Chu et al., 2003; Seebohm and Schreiber, 2021). Thus, CML cells were treated with CPS (10–300 μM) and OLDA (0.5–100 μM) for 24 h and analyzed by cell viability assay. OLDA induced a stronger decrease in cell viability than CPS, as shown by the IC_50_ values: 14.1 μM vs 140.1 μM in K562, 1.4 μM vs 129.8 μM in KU812, and 8.4 μM vs 173.9 μM in MOLM-6 (Figures 1A, B). For this reason, we selected OLDA as the TRPV1 agonist, and doses in the IC_50_ range (10 µM for K562 and MOLM-6; 1 µM for KU812) are considered for the subsequent experiments.

To assess the involvement of TRPV1, cell viability assay was evaluated in CML cells silenced for TRPV1 expression (siTRPV1) (Supplementary Figures S2A, B). TRPV1 silencing significantly reduced the OLDA effects compared to siGLO control cells, confirming that OLDA decreases cell viability by activating this channel (Figure 1C). We also assessed cell viability in normal myeloid cells, enriched from the blood of healthy donors, treated or not treated with OLDA at 1 µM and 10 µM (Supplementary Figure S3A). Results showed that in normal mature myeloid cells, OLDA is much less effective at reducing growth. This result is in line with our *in silico* analysis, in which the downregulation of TRPV1 expression occurs along the differentiation process (Supplementary Figure S3B).

### 3.2 The triggering of TRPV1 by OLDA induces oxidative stress in CML cells

**FIGURE 2:**
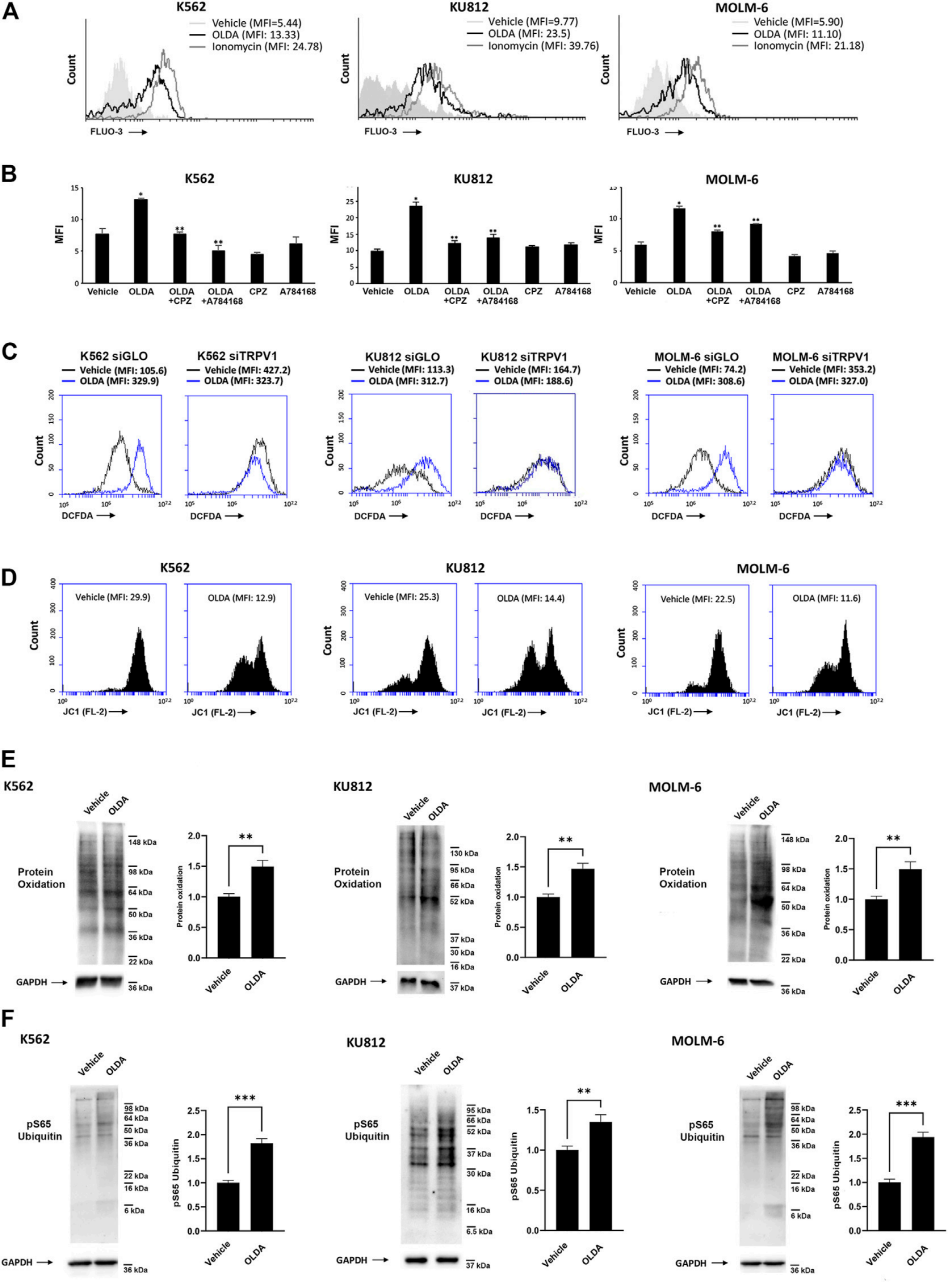
Triggering of TRPV1 stimulates [Ca^2+^]_i_ and oxidative stress in CML cells. **(A)** [Ca^2+^]_i_ evaluated by Fluo-3 staining and flow cytometric analysis in CML cells treated for 1 min with OLDA (IC_50_ dose). MFI, mean fluorescence intensity. **(B)** [Ca^2+^]_i_ assessed by Fluo-3 staining and flow cytometric analysis in CML cells pretreated with CPZ or A784168 for 3 min before the addition of OLDA for 1 min. Data are the mean ± SD of three experiments. *p < 0.05 vs vehicle; **p < 0.05 vs OLDA-treated cells. **(C)** ROS were investigated by DCFDA staining and flow cytometric analysis in siGLO and siTRPV1 CML cells treated with the vehicle or OLDA (IC_50_ dose) for 6 h. MFI, mean fluorescence intensity. Data are representative of one out of three separate experiments. **(D)** ΔΨm changes in OLDA-treated CML cells by JC-1 staining and flow cytometric analysis. Drop in ∆Ψm decreases the J-aggregate (red fluorescence). MFI, mean fluorescence intensity. Data are representative of three experiments. **(E, F)** Western blot analysis was performed on lysates from CML cells treated with OLDA or the vehicle for 24 h to investigate protein oxidation **(E)** and pSer65 ubiquitin **(F)**. Blots are representative of three experiments. GAPDH was used as the loading control. Folds (mean ± SD of three experiments) = changes compared to the vehicle. **p < 0.01; ***p < 0.001.

The opening of the TRPV1 ligand-gated ion channel promotes transmembrane Ca^2+^ entry, which affects the fine balance between death and survival signaling pathways (Zhai et al., 2020). To assess the signaling pathway induced by OLDA, we first performed calcium mobilization assay for up to 3 min (data not shown). The increase in [Ca^2+^]_i_ was found in CML cells treated with OLDA for 1 min after the stimulation compared to the vehicle (Figure 2A). This effect was inhibited by both the TRPV1 antagonists CPZ and A784168, validating the TRPV1 involvement in OLDA-induced effects (Figure 2B). Moreover, the [Ca^2+^]_i_ overload was associated with a clear rise in ROS production induced by OLDA in a TRPV1-dependent manner as shown by the evident increase in DCFDA fluorescence in siGLO but not in siTRPV1 CML cells (Figure 2C; Supplementary Figure S4A). Mitochondria dysfunction is often the consequence of [Ca^2+^]_i_ influx and ROS accumulation (Brookes et al., 2004). Thus, by performing JC-1 staining and FACS analysis, we showed that the treatment of CML cells with OLDA for 24 h induces a marked reduction in JC-1 red fluorescence, demonstrating mitochondrial depolarization (Figure 2D; Supplementary Figure S4B). Given that the interplay between [Ca^2+^]_i_ and ROS, leading to mitochondrial impairment, is responsible for changes in macromolecules with consequent alterations of the cellular redox state (Feno et al., 2019), Western blot analysis was performed to better elucidate the oxidation levels. Our results showed that OLDA treatment induces a robust enhancement in the oxidation of total proteins in all CML cell lines (Figure 2E). In addition, we also found that phospho-ubiquitin (Ser65) levels, correlated with injured mitochondria (Hepowit et al., 2022), are strongly enhanced in OLDA-treated CML cells (Figure 2F), supporting that OLDA, by triggering TRPV1, induces oxidative stress and mitochondrial dysfunction.

### 3.3 OLDA treatment, *via* TRPV1, induces ER stress but not autophagy in CML cells

**FIGURE 3:**
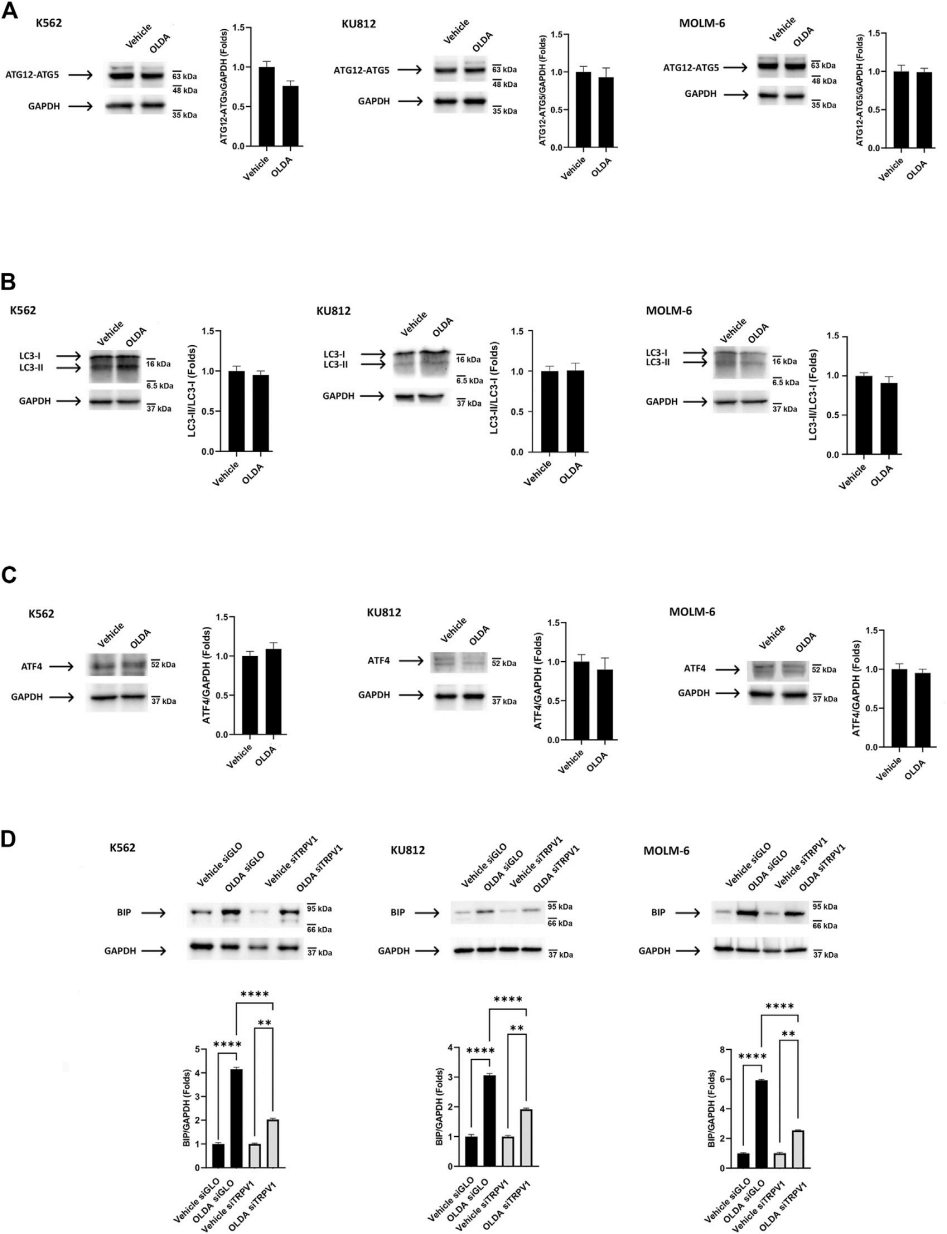
TRPV1 activation by OLDA promotes ER stress in CML cells. **(A, B)** Western blot analysis of the ATG12–ATG5 complex **(A)**, LC-3 II **(B)**, and ATF4 **(C)** in CML cells treated with OLDA at the IC_50_ dose for 24 h. **(D)** Western blot analysis of BiP in siGLO and siTRPV1 CML cells treated as previously described. Blots are representative of three experiments. GAPDH was used as the loading control. Folds (mean ± SD of three experiments) = changes compared to the vehicle. **p < 0.01; ****p < 0.0001.

Autophagy, activated in response to several conditions including oxidative stress and mitochondrial alteration, is an essential pathway aimed at eliminating unwanted intracellular elements, such as unfolded oxidized proteins or damaged organelles, to promote cell survival (García Ruiz et al., 2022). To assess the induction of autophagy by OLDA treatment, we first analyzed the ATG12–ATG5 complex essential for autophagosome formation (Kharaziha and Panaretakis, 2017). We found that in OLDA-treated CML cells, no increase in the expression levels of ATG12–ATG5 complex was evident, supporting that the execution of autophagy is not stimulated (Figure 3A). In addition, the conversion of the soluble form of LC3-I to the lipidated and autophagosome-associated form (LC3-II) was investigated. We found that OLDA treatment was not able to increase the expression of LC3-II in all CML cell lines (Figure 3B). To further strengthen our data, we also investigated the expression of ATF4, a transcription factor involved in stress-induced autophagy gene expression (B’chir et al., 2013). We showed that the treatment with OLDA was not able to upregulate the expression of ATF4 compared to vehicle-treated CML cells, highlighting once again that autophagy is not activated (Figure 3C).

These results prompted us to investigate endoplasmic reticulum stress, found to be stimulated under cellular stress conditions, which is characterized by the accumulation of misfolded proteins detected by the unfolded protein response (UPR). BiP, a chaperone protein abundant in ER, is considered the master stress sensor involved in UPR activation (Kopp et al., 2019). Therefore, to investigate the ER stress induced by OLDA *via* TRPV1, CML cells silenced for TRPV1 expression were treated with OLDA for 24 h. We found that the treatment with OLDA induces a strong upregulation of BiP expression levels in siGLO CML cells, used as the control, compared to siTRPV1 CML cells, indicating that ER stress is activated in a TRPV1-dependent manner (Figure 3D).

### 3.4 The ER stress induced by OLDA treatment leads to apoptotic cell death

**FIGURE 4:**
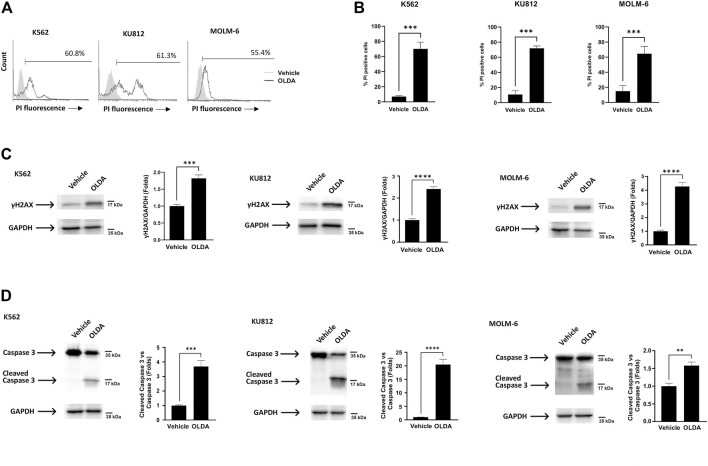
Triggering of TRPV1 promotes apoptotic cell death of CML cells. **(A)** PI staining of CML cells treated with OLDA at the IC_50_ dose or with the vehicle for 24 h. Data are representative of three experiments. Percentage indicates PI-positive cells. **(B)** Percentage of PI-positive cells in CML cells treated as previously described. Data are the mean ± SD of three separate experiments. ***p < 0.001. **(C, D)** Western blot analysis of γH2AX **(C)** and caspase-3 cleavage **(D)** in CML cells treated for 24 h with OLDA at the IC_50_ dose. Blots are representative of three experiments. GAPDH was used as the loading control. Folds (mean ± SD of three experiments) = changes compared to the vehicle. **p < 0.01; ***p < 0.001; ****p < 0.0001.

Our next step was to investigate cell death in CML cells treated with the TRPV1 agonist OLDA, given that the UPR pathway may play a dual role in either providing survival benefits or triggering cell death (Mlynarczyk and Fåhraeus, 2014). The cytofluorimetric analysis showed that OLDA enhances the percentage of PI fluorescent cells, indicating the activation of cell death (Figures 4A, B), and, moreover, upregulates the expression of the Ser139-phosphorylated variant of histone 2A (γH2AX), supporting the presence of DNA double-strand breaks (Figure 4C). Finally, to assess the type of cell death, the analysis of caspase-3 cleavage was performed. The presence of cleaved caspase-3 fragments in OLDA-treated CML cells was found, demonstrating that the oxidative stress induced *via* TRPV1 by OLDA promotes apoptotic cell death in CML cells (Figure 4D).

### 3.5 OLDA synergizes with imatinib in reducing cell viability of CML cells

**FIGURE 5:**
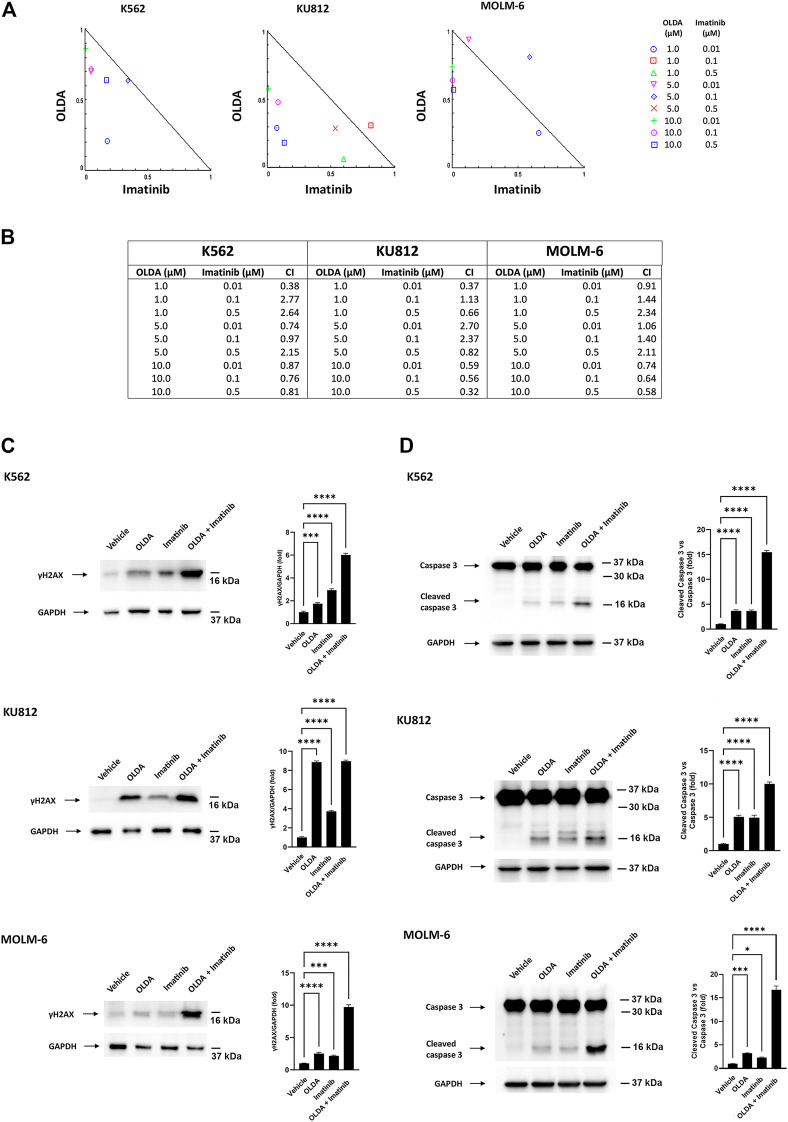
OLDA and imatinib synergize in reducing cell viability. **(A)** Isobologram plots for combination treatments of OLDA and imatinib. Lower left of the hypotenuse, synergism; hypotenuse, additive effect; upper right of the hypotenuse, antagonism. Data are representative of three experiments. **(B)** CI values obtained using CompuSyn software. Data are representative of three experiments. **(C, D)** Western blot analysis of γH2AX **(C)** and caspase-3 cleavage **(D)** in CML cells treated for 24 h with the vehicle or with OLDA at the IC_50_ dose or imatinib (0.5 μM), alone or in combination. Blots are representative of three experiments. GAPDH was used as the loading control. Folds (mean ± SD of three experiments) = changes compared to the vehicle. *p < 0.05; ***p < 0.001; ****p < 0.0001.

Presently, the common therapeutic strategy for the treatment of CML is based on TKIs such as imatinib. Therefore, as new strategies and new drug targets are always embraced to increase treatment possibilities, we used a computational approach to analyze the experimental data in order to elucidate the nature of the interaction between OLDA and imatinib. Cells were exposed to different doses of OLDA and imatinib for 24 h, and then the cell viability test was performed. Isobologram analysis demonstrated that several combinations of the two drugs provoke increased levels of cytotoxicity, when compared to single treatments (Figure 5A). In particular, the CI values obtained by combining OLDA at the IC_50_ dose with all the three doses of imatinib are < 1, indicating synergistic effects (Figure 5B). To support these data, we also performed Western blot analysis to assess DNA damage and apoptosis on CML cells treated with the combination of OLDA (IC_50_) and imatinib (0.5 µM). Our data demonstrated that the co-administration of both drugs is more effective in inducing upregulation of γH2AX and increasing caspase-3 fragment levels than the single treatment (Figures 5C, D), confirming the synergistic effects.

## 4 Discussion

As a result of an imbalance between proliferation and apoptosis with a marked preference for cell growth, cancer is characterized by a decrease in apoptosis, leaving malignant cells resistant to death (Wong, 2011). However, apoptosis presents itself as a double-edged sword. In fact, it can be the problem and also provide a potential solution, since drugs aimed at activating and/or increasing apoptotic pathways are indeed coveted therapeutic options, especially in the field of leukemia (Peng et al., 2016; Bulut et al., 2022). In this scenario, Ca^2+^ influx is the master second messenger that influences the proliferation–apoptosis balance: low intracellular levels are required for cell growth stimulation, while its overload is strongly responsible for apoptosis induction (Shapovalov et al., 2011). Given that many anti-cancer drugs induce reduction in cell growth by disturbing [Ca^2+^]_i_, ion channels are interesting targets for therapy. In this regard, TRPV1, a ligand-gated cation channel also known as the CPS receptor with selectivity for Ca^2+^ over Na^+^, in addition to regulating nociception, metabolism, and thermoregulation, is involved in the modulation of the proliferation–apoptosis equilibrium, exactly because it modifies [Ca^2+^]_i_. It is expressed in neuronal and non-neuronal healthy cells as well as in many different cancer cells including acute T-cell leukemia (Zhai et al., 2020). Our previous work (Maggi et al., 2022) demonstrated that TRPV1 was expressed at the molecular and protein levels in several CML cell lines. Furthermore, this study strengthened the evidence of expression of TRPV1 in all leukemia types, emphasizing its modulation during the progression of the disease with higher levels of leukemic common progenitors during the chronic phase where additional epigenetic changes contribute to blastic transformation and TKI resistance. In fact, patients with chronic-stage CML have many CD34^+^ leukemia cells with innate resistance to imatinib at the time of diagnosis (Perrotti et al., 2010; Pascu VÎnturiŞ and GĂman, 2020).

It is well known that TRPV1 activation in cancer cells such as urothelial, endometrial, and colorectal cancer cells, by strongly enhancing [Ca^2+^]_i_, promotes mitochondrial depolarization and ROS overproduction that leads to apoptotic cell death (Amantini et al., 2009; Fonseca et al., 2018; Hou et al., 2019). In agreement with these findings, we demonstrated that the triggering of TRPV1, by its agonist OLDA, induces increase in Ca^2+^ influx and ROS production with consequent mitochondrial dysfunction. Interestingly, the induction of mitochondrial impairment represents a promising approach given that the increased number of mitochondria and exaggerated mitochondrial activity were recognized in leukemia cells compared with normal hematopoietic stem cells (Sriskanthadevan et al., 2015; Barbato et al., 2020). Our data also showed that the increase in ROS, associated with the induction of oxidative stress and subsequent accumulation of oxidized proteins, does not promote survival autophagy but stimulates, in a TRPV1-dependent manner, ER stress followed by apoptosis. The autophagy machinery is activated during cellular stresses to digest damaged organelles, unwanted proteins, and intracellular materials. Thus, given that it is indispensable to maintain cellular homeostasis, several reports highlight its anti-tumoral action in CML by controlling ROS and DNA damage (Baquero et al., 2019; García Ruiz et al., 2022). It has been demonstrated that CD34^+^ leukemia stem cells display a high level of autophagic flux, and, more importantly, TKI treatment, as the flip side, stimulates autophagy responsible for leukemia stem cell survival and establishment of drug resistance (Bellodi et al., 2009). In fact, the targeting of autophagy with inhibitors in combination with conventional therapy has been suggested in the CML treatment, also to counteract the BCR-ABL-independent mechanism of resistance (Mitchell et al., 2018). Thus, our results indicate that the induction of apoptosis, by the triggering of TRPV1, without stimulation of autophagic signals, could represent a good opportunity to contrast the survival signals induced by the same TKI therapy.

To date, much effort has been made to understand the role of ROS in CML pathogenesis. It is clear that leukemia progenitor cells are characterized by higher levels of ROS than normal hematopoietic cells. BCR-ABL1 oncoprotein is involved in the production of ROS that are then responsible for promoting genomic instability, a sort of “auto-mutagenesis” process that leads to malignancy (Pascu VÎnturiŞ and GĂman, 2020; Głowacki et al., 2021). In addition, it has surprisingly been found that TKI treatment during time favors the control of the redox cell state by activating the repair enzyme human MutT homolog 1 which removes the oxidatively damaged cellular nucleotide pool and repairs the DNA breaks, leading to a strong reduction in therapy efficacy itself and chemoresistance (Lee et al., 2022). On the other hand, the drug-induced oxidative stress exaggeration in leukemic cells could intensify the genotoxic effects of conventional therapy, facilitating the activation of programmed cell death (Yang et al., 2018). In fact, recent findings showed that the stimulation of oxidative stress in CML, using compounds such as tyrosol derivatives, verbascoside, and zerumbone, promotes mitochondrial membrane potential dissipation, caspase activation, and apoptosis (Rajan et al., 2015; Abdelkafi-Koubaa et al., 2022; Akgun-Cagliyan et al., 2022). Therefore, the strategy of deliberately increasing ROS to cytotoxic levels for leukemia cells seems to be very appealing, and our data are in agreement. The oxidative stress is often followed by the accumulation of unwanted proteins and ER stress, which triggers UPR to restore the homeostatic state, when the condition is toxic for the cell, and promote cell death (Kopp et al., 2019). Accordingly, our data consistently demonstrate that ER stress is activated by TRPV1, leading to apoptotic cell death. In this regard, ER stress-induced apoptosis of leukemia cells was also demonstrated (Storniolo et al., 2015). Therefore, our results, by determining that the TRPV1-induced signaling cascade is responsible for increased ROS, ER stress, DNA damage, and programmed cell death, are in line with these findings. Moreover, we described a synergistic effect induced by combining the TRPV1 activator OLDA with imatinib in stimulating both DNA breaks and apoptosis in CML. This highlights the possibility to use TRPV1 triggering to counteract negative side of the TKI therapy itself and limit the development of resistance.

However, even if the signaling pathways induced by the TRPV1 activation were elucidated in three different CML cell lines, our study is based on an *in vitro* model. Thus, to bridge the gaps, our future direction is to confirm our results by using both blood collected from CML patients stratified according to TKI resistance and mouse models of CML. In recent years, several *in vivo* models of CML have been established, by using chimeric mouse strains as well as different immunocompromised strains (Sontakke et al., 2016), to study the molecular pathogenesis of the disease and approach new therapeutic strategies. Thus, transgenic, conditioned, and/or xenograft mice CML models can be used to validate the effects of TRPV1 activation triggered by OLDA.

Interesting and innovative oncologic approaches based on TRPV1 activation have been developed in anti-cancer research, such as photothermal stimulation, development of specific nano-agonists, gold nanorod-assisted near-infrared irradiation-activated tool, and many synthetic as well as natural-origin activators. The aim of these studies is to exploit the opening of the TRPV1 channel to increase the cytoplasmic Ca^2+^ flux leading to apoptosis (Zhai et al., 2020). Overall, our findings underline the importance to trigger TRPV1 as an important avenue in CML management.

## Data Availability

The raw data supporting the conclusion of this article will be made available by the authors, without undue reservation.
