# Supplementary material for: Calcium influx, oxidative stress, and apoptosis induced by TRPV1 in chronic myeloid leukemia cells: Synergistic effects with imatinib
Source: Front Mol Biosci. 2023 Feb 15;10:1129202. doi: 10.3389/fmolb.2023.1129202 (PMC9975599; doi:10.3389/fmolb.2023.1129202)
Supplement: Supplementary file 1 [file DataSheet1.PDF]

Supplementary Material

Calcium influx, oxidative stress and apoptosis induced by TRPV1 in chronic myeloid leukemia cells: synergistic effects with Imatinib

Federica Maggi, Maria Beatrice Morelli, Cristina Aguzzi, Laura Zeppa, Massimo Nabissi, Carlo Polidori, Giorgio Santoni and Consuelo Amantini*

*** Correspondence:** Consuelo Amantini: consuelo.amantini@unicam.it

Supplementary Figures

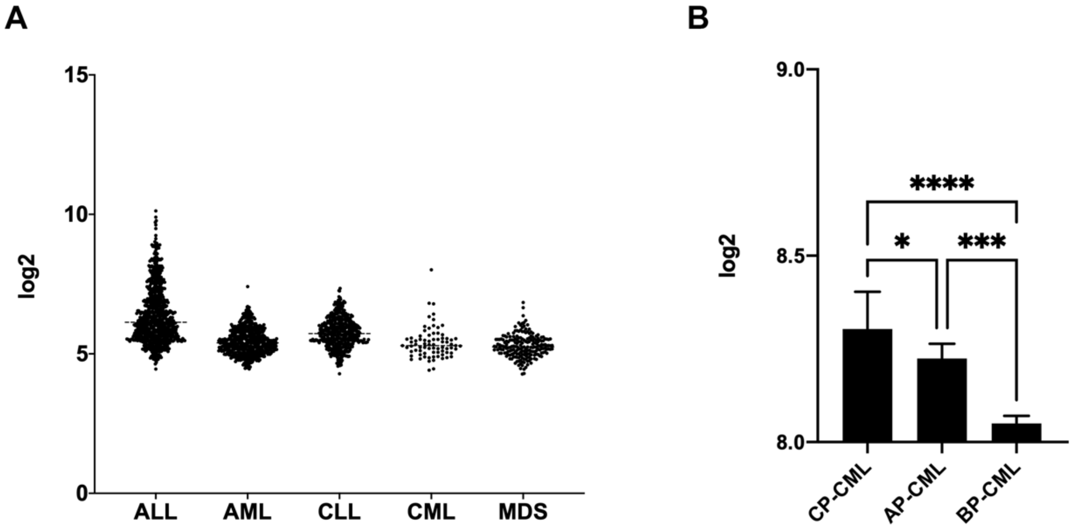

Supplementary Figure 1. TRPV1 expression in leukemia. A) *In silico* analysis of data from GEO to evaluate the expression of TRPV1 in leukemia patients. ALL: acute lymphoblastic leukemia; AML: acute myeloid leukemia; CLL: chronic lymphoblastic leukemia; CML: chronic myeloid leukemia; MDS: myeloproliferative disorders. B) *In silico* analysis of data from Stemformatics to assess the

expression of TRPV1 in patients with CML in different stages. CP: chronic phase; AP: accelerated phase; BP: blastic phase. *p<0.05; ***p<0.001; ****p<0.0001.

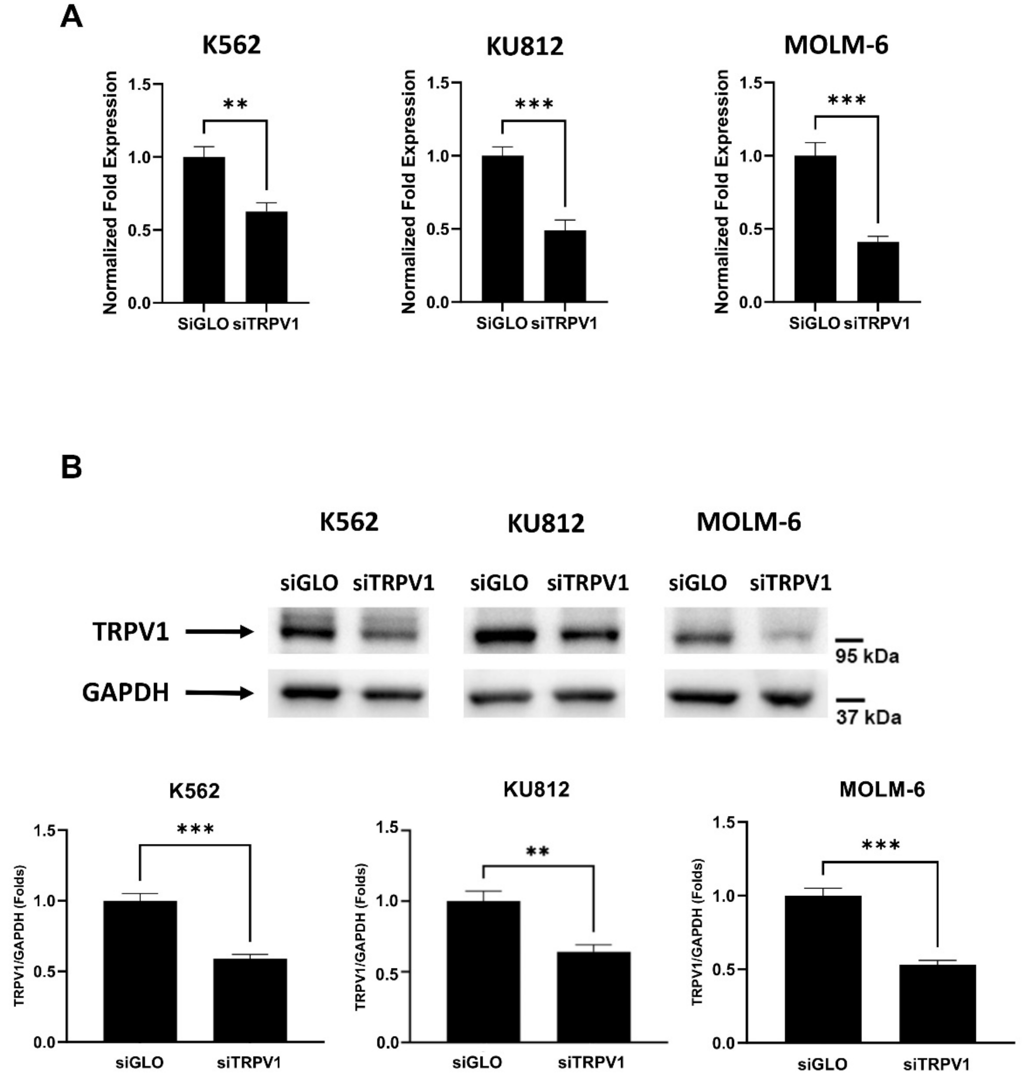

Supplementary Figure 2. TRPV1 silencing. A) The efficiency of TRPV1 silencing was evaluated by qRT-PCR. mRNA levels were normalized for GAPDH expression. Data, the mean ± SD of three separate experiments, are expressed as fold changes respect to siGLO. **p<0.01; ***p < 0.001 B) TRPV1 protein downregulation assessed by western blot. Immunoblots are representative of separate experiments. GAPDH was used as loading control. Data of the densitometric analysis are the mean ±

SD of three different experiments and are expressed as fold by using siGLO as control. **p<0.01; ***p < 0.001.

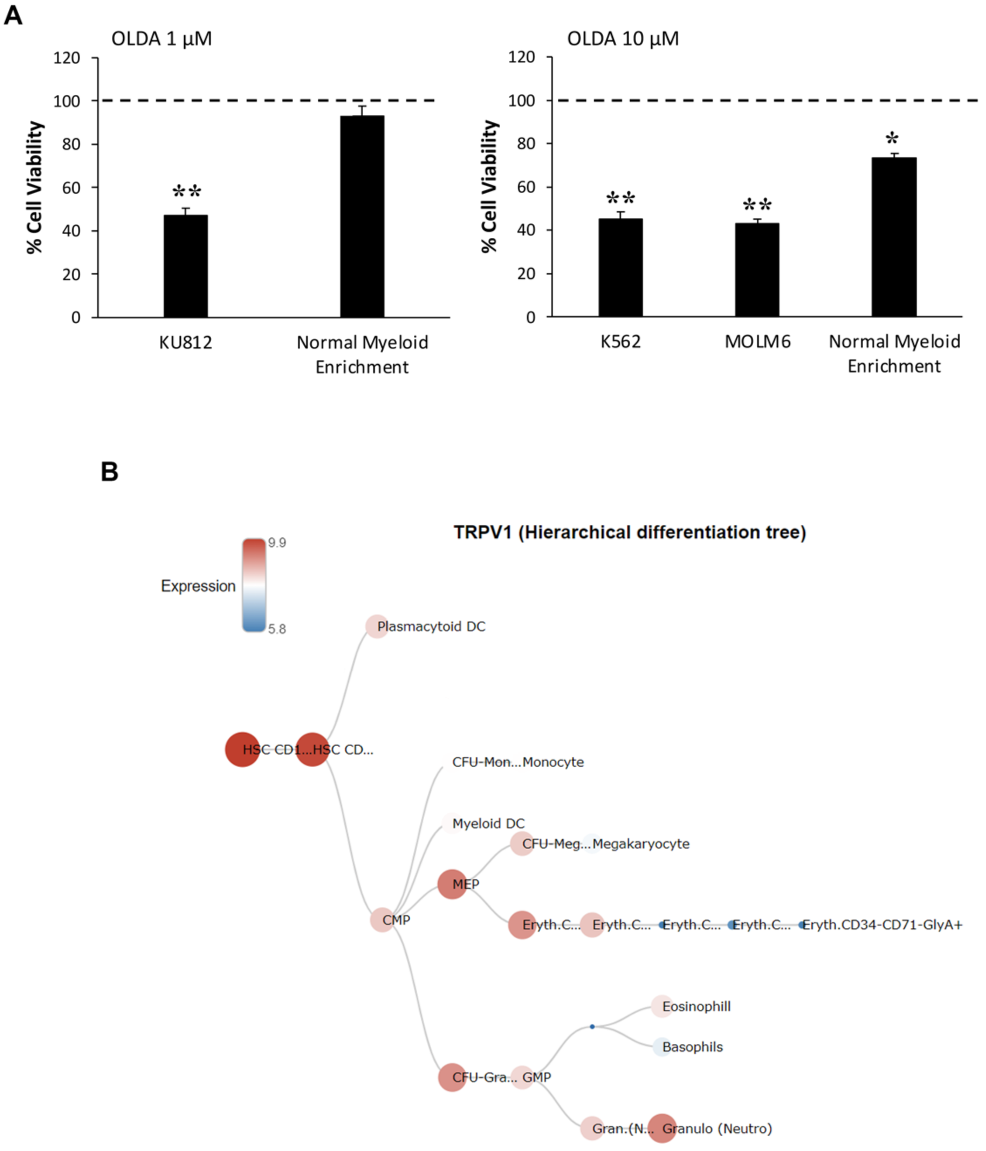

Supplementary Figure 3. The TRPV1 agonist OLDA is less effective in normal mature myeloid cells with respect to CML cells. A) Cell viability was performed in CML cells and in myeloid enrichment from healthy blood donors treated with vehicle or with OLDA at IC_{50} doses. *p<0.05 vs vehicle; **p<0.01 vs OLDA-treated CML cells. B) Hierarchical differentiation tree showing the TRPV1 expression in normal myeloid cells during maturation.

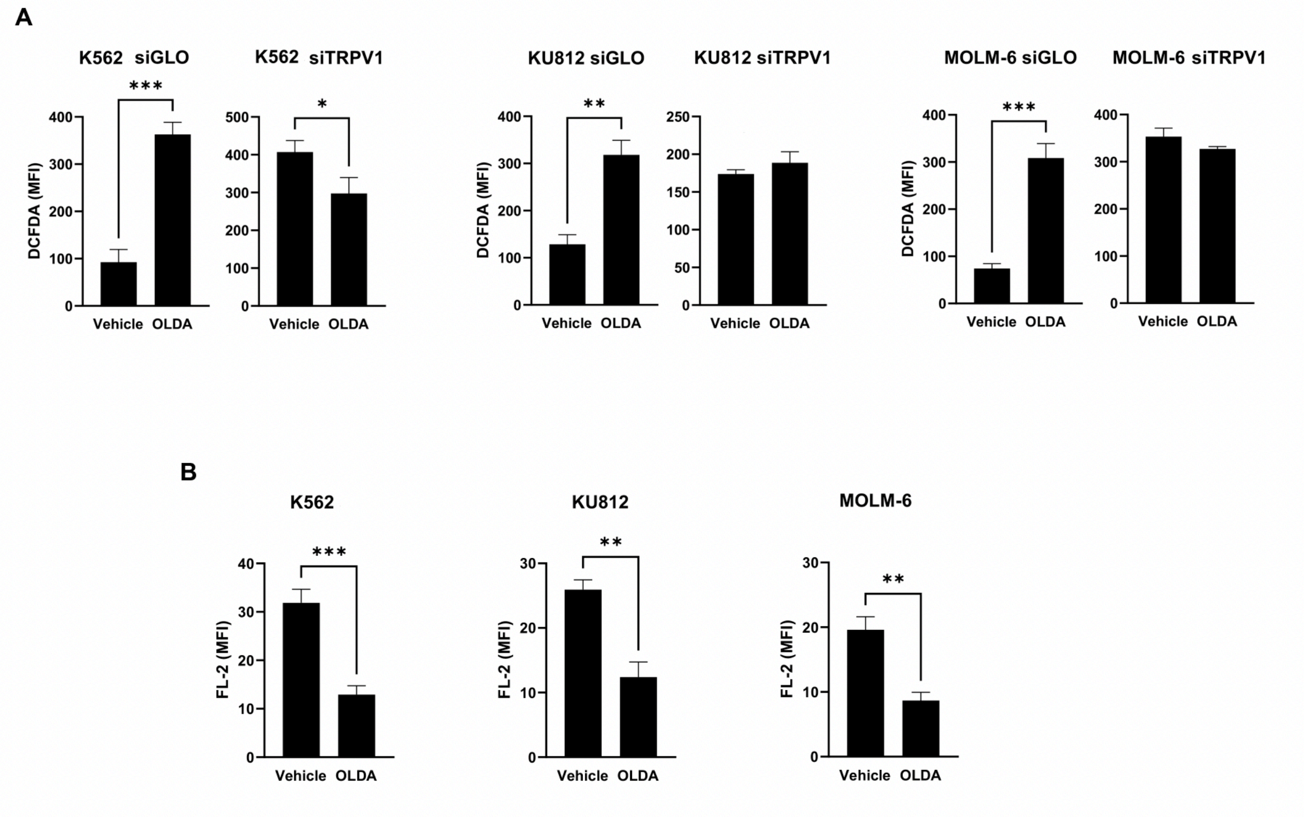

Supplementary Figure 4. The triggering of TRPV1 induces oxidative stress in CML cells. A) Quantification of ROS level presented as change of mean fluorescence intensity (MFI) in OLDA-treated siGLO and siTRPV1 CML cells with respect to their controls. Data are the mean ± SD of three experiments. *p<0.05; **p<0.01; ***p<0.001. B) Drop of ΔΨm evaluated by JC-1 in OLDA-treated CML cells. Data are the mean ± SD of three experiments **p<0.01***p<0.001. MFI: Mean Fluorescence Intensity.
